# Supplementary material for: Resolving the full spectrum of human genome variation using Linked-Reads
Source: Genome Res. 2019 Apr;29(4):635–45. doi: 10.1101/gr.234443.118 (PMC6442396; doi:10.1101/gr.234443.118)
Supplement: Supplemental Material [file supp_gr.234443.118_Supplemental_longranger-2.2.zip › longranger-2.2.tar.bz2!/lr2.2-public/lib/stan/cmdstan/stan/doc/api/html/_eigen___num_traits_8hpp.html]

Stan: src/stan/agrad/rev/matrix/Eigen\_NumTraits.hpp File Reference


|  |  |
| --- | --- |
|  | Stan  2.5.0  probability, sampling & optimization |


- Main Page
- Related Pages
- Namespaces
- Classes
- Files

- File List
- File Members

All Classes Namespaces Files Functions Variables Typedefs Enumerations Enumerator Friends Macros Pages

- src
- stan
- agrad
- rev
- matrix

Classes |
Namespaces

Eigen\_NumTraits.hpp File Reference

`#include <limits>`  
`#include <stan/math/matrix/Eigen.hpp>`  
`#include <stan/agrad/rev/var.hpp>`

Go to the source code of this file.

|  |  |
| --- | --- |
| Classes | |
| struct | Eigen::NumTraits< stan::agrad::var > |
|  | Numerical traits template override for Eigen for automatic gradient variables. More... |
|  | |
| struct | Eigen::internal::significant\_decimals\_default\_impl< stan::agrad::var, false > |
|  | Implemented this for printing to stream. More... |
|  | |
| struct | Eigen::internal::scalar\_product\_traits< stan::agrad::var, double > |
|  | Scalar product traits override for Eigen for automatic gradient variables. More... |
|  | |
| struct | Eigen::internal::scalar\_product\_traits< double, stan::agrad::var > |
|  | Scalar product traits override for Eigen for automatic gradient variables. More... |
|  | |
| struct | Eigen::internal::general\_matrix\_vector\_product< Index, stan::agrad::var, ColMajor, ConjugateLhs, stan::agrad::var, ConjugateRhs > |
|  | Override matrix-vector and matrix-matrix products to use more efficient implementation. More... |
|  | |
| struct | Eigen::internal::general\_matrix\_vector\_product< Index, stan::agrad::var, RowMajor, ConjugateLhs, stan::agrad::var, ConjugateRhs > |
|  | |
| struct | Eigen::internal::general\_matrix\_matrix\_product< Index, stan::agrad::var, LhsStorageOrder, ConjugateLhs, stan::agrad::var, RhsStorageOrder, ConjugateRhs, ColMajor > |
|  | |

|  |  |
| --- | --- |
| Namespaces | |
|  | stan |
|  | Probability, optimization and sampling library. |
|  | |
|  | stan::agrad |
|  | Function gradients via reverse-mode automatic differentiation. |
|  | |
|  | Eigen |
|  | (Expert) Numerical traits for algorithmic differentiation variables. |
|  | |
|  | Eigen::internal |
|  | (Expert) Product traits for algorithmic differentiation variables. |
|  | |

## Variable Documentation

stan::agrad::vari\* alpha\_

Definition at line 14 of file Eigen\_NumTraits.hpp.

double dotval\_

Definition at line 17 of file Eigen\_NumTraits.hpp.

size\_t length\_

Definition at line 18 of file Eigen\_NumTraits.hpp.

stan::agrad::vari\*\* v1\_

Definition at line 15 of file Eigen\_NumTraits.hpp.

stan::agrad::vari\*\* v2\_

Definition at line 16 of file Eigen\_NumTraits.hpp.


---

[ Stan Home Page ]

*© 2011–2014,
Stan Development Team.*
